# BMC Biology turns five

**DOI:** 10.1186/1741-7007-6-53

**Published:** 2008-12-16

**Authors:** Elizabeth C Moylan, Matt J Hodgkinson, Maria Kowalczuk, Scott C Edmunds, Penelope A Webb

**Affiliations:** 1 BioMed Central, Middlesex House, 34-42 Cleveland St., London, W1T 4LB, UK

*BMC Biology *launched in November 2003, under the stewardship of Peter Newmark and an international Editorial Board [1] as the flagship open access biology journal in the *BMC *series, publishing research of general interest and special importance across the biological sciences. The aim was to bridge a gap between the premier journal, *Journal of Biology *[2] a home for exceptional research, and the established specialist titles in the *BMC *series [3], such as *BMC Bioinformatics*, by providing a more selective home for articles of broader interest. As *BMC Biology*'s fifth birthday is upon us, it has secured its position within the *BMC-series *stable with an impressive debut impact factor of, appropriately, five!

We are absolutely delighted with our impact factor of five, but how was this achieved? One clear contributor to the impact factor is from the field of plant genomics, a paper by Christopher Town and colleagues (*Complete reannotation of the *Arabidopsis *genome: methods, tools, protocols and the final release*) [4] that has been cited over 50 times and is the most highly cited article published in *BMC Biology*. This is closely followed by two papers on species boundaries, from the research groups of Brian G Spratt (*Fuzzy species among recombinogenic bacteria*) [5] and James Mallet (*Polyphyly and gene flow between non-sibling *Heliconius *species*) [6]. Most of our published articles are from evolutionary biology, cell biology and neuroscience with genomics and developmental biology hot on their heels [7], although we welcome submissions across the full spectrum of biology.

Feedback from our authors makes it clear that they value the 'added extras' that publishing in *BMC Biology *brings. *Faculty of 1000*, a research service that highlights the most interesting papers published in the biological sciences [8], regularly features our research articles. Our sister journal, *Journal of Biology*, often publishes minireviews that put the work published in *BMC Biology *into a broader context, further widening the readership of the original research papers [2]. Most important, many of our research articles are press released [9] and generate considerable media interest. A notable example was a correspondence article by Martin Collinson on a video analysis of a Pileated Woodpecker that called into question the apparent sighting of the seemingly extinct Ivory-billed Woodpecker *Campephilus principalis *[10]. Finally, all articles are featured with a summary on the *BMC Biology *homepage, and many on the BioMed Central homepage.

It is gratifying to see that on the strength of its diverse and high-quality content over the last five years, *BMC Biology *has been ranked 219 of nearly 16,000 journals listed in the 2007 SCImago Journal Rank [11], a journal citation metric derived from Scopus [12]. This places *BMC Biology *in the top 1.5% of all journals. If you value the benefits brought by publishing in a high quality open access journal, with a dedicated editorial team, and a rigorous but fair and friendly peer review service, we look forward to receiving your next submission to *BMC Biology*.
